# Oxidative stress and NF-KB/iNOS inflammatory pathway as innovative biomarkers for diagnosis of drowning and differentiating it from postmortem submersion in both fresh and saltwater in rats

**DOI:** 10.1007/s00414-024-03249-5

**Published:** 2024-05-27

**Authors:** Rana Adel, Manar Fouli Gaber Ibrahim, Samar Hisham Elsayed, Nada A. Yousri

**Affiliations:** 1 Forensic Medicine and Clinical Toxicology Department, Faculty of Medicine, Minia University, Minya, Egypt; 2 Histology and Cell Biology Department, Faculty of Medicine, Minia University, Minya, Egypt; 3 Medical Biochemistry Department – Faculty of Medicine, Minia University, Minya, Egypt

**Keywords:** Drowning, Freshwater, Saltwater, Acute lung injury, Postmortem submersion

## Abstract

**Background:**

Finding a dead body in water raises an issue concerning determining the cause of death as drowning because of the complex pathophysiology of drowning. In addition, the corpse may be submersed postmortem.

**Objective:**

Evaluate the role of oxidative stress markers and NF-KB/iNOS inflammatory pathway as diagnostic biomarkers in drowning and whether they could differentiate freshwater from saltwater drowning.

**Methods:**

This study included forty-five adult male albino rats classified into five groups: control group (C), Freshwater-drowned group (FD), Freshwater postmortem submersion group (FPS), saltwater-drowned group (SD), and saltwater postmortem submersion group (SPS). After the autopsy, the rats' lungs in each group were prepared for histological, immunohistochemical (caspase 3, TNF-α, NF-kB, COX-2 & iNOS), biochemical studies; MDA, NOx, SOD, GSH, VCAM-1, COX-2; and RT-PCR for the relative quantification of NF-kB and iNOS genes expression.

**Results:**

Lung oxidative markers were significantly affected in drowned groups than in postmortem submersion groups. Inflammatory pathway markers were also significantly increased in the drowned groups, with concern that all markers were significantly affected more in saltwater than in freshwater drowned group.

**Conclusions:**

It is concluded that the tested markers can be used accurately in diagnosing drowning and differentiating it from postmortem submersion with a better understanding of the mechanism of death in drowning as both mechanisms, inflammatory and oxidative stress, were revealed and involved.

## Introduction

Aspiration of fluid into the airways causes drowning, which in turn causes suffocation, vagus nerve inhibition, electrolyte imbalances, laryngospasm, hypothermia, and secondary damage [1]. Drowning's pathophysiological mechanisms are convoluted, incompletely understood, and characterised by speculation at best [2]. When a person drowns, the lung is the primary organ to fail [3]. Drowning victims hold their breath and develop hypercarbia and hypoxemia; they then rebreathe and begin to inhale water, which causes lung damage and provokes ventilation-perfusion mismatch, hypoxia, and mitochondrial malfunction; these are the gravest sequels of drowning and are linked to the onset of acute lung injury [4, 5].

Diagnosis of drowning is a challenging job in forensic medicine; additionally, identifying a drowned body from a corpse placed into the water after decease is an obstacle for forensic pathologists [6]. Drowning is usually diagnosed based on a number of factors, including the scene, a complete autopsy, histological results, and a diatom test; nevertheless, these factors provide only circumstantial and not convincing proof for the diagnosis [7].

Many studies depend on histological methods to diagnose drowning and provide information about the pathophysiology of freshwater and saltwater drowning as Barranco et al. [8] evaluated the lung tissue morphometry changes in freshwater and saltwater drowning using optic microscopy and found that freshwater drowning is characterized by aquatic emphysema.

Nowadays, various molecular biological methods are frequently used and may guarantee the objectivity and precision of forensic investigations. Recent researchers have investigated the biological role of different molecules in different tissues, including aquaporin, inflammatory cytokines, and surfactant–protein, that could be valuable in the diagnosis of drowning [9, 10], for instance, Barranco et al. [11] studied renal expression of aquaporin 2, arginine-vasopressin, vasopressin receptor 2, in diagnosing drowning and concluded that arginine-vasopressin and aquaporin 2 were helpful in diagnosis and might differentiate freshwater from saltwater drowning. However, the majority of these researches either failed to distinguish between drowning and postmortem submersion or only examined seawater aspiration [1]. Therefore, this study aimed to investigate the role of oxidative stress biomarkers and NF-KB/iNOS inflammatory pathway as diagnostic biomarkers in drowning and whether they could differentiate freshwater from saltwater drowning.

## Materials and methods

### Animals

Forty-five albino rats (adult male, 250–300 g and 12–16 weeks old) were acquired from the experimental animals growing centre at Minia University. The rats were acclimated for two weeks in a well-ventilated laboratory with regulated temperature (22 ± 2 ºC) and lighting (12-h light/dark cycle) in standard cages provided with ad libitum standard diet feeding and tap water drinking.

### Ethical approval

The experiment was done in compliance with the ethical committee's recommendations and guidelines for animal use and care at Minia University, approval No. 814:6:2023.

### Water sample collection

We collected the experiment's freshwater from the Nile River near Minia Governorate in Egypt (salinity of 0.5 per cent) [12] and the experiment's saltwater from the Red Sea near Hurghada Town (salinity of 40.6 per cent) [13].

### Experimental design

We randomly allocated the rats into five groups, nine rats per group: control group (C); Freshwater-drowned group (FD); Freshwater postmortem submersion group (FPS); saltwater-drowned group (SD); saltwater postmortem submersion group (SPS).

### Control group (C)

Rats were anaesthetised with 40 mg/kg body weight Na thiopental injected intraperitoneally, and then the sacrifice was made by cervical dislocation.

### Freshwater-drowned group (FD) and saltwater-drowned group (SD)

To imitate actual drowning, rats were injected intraperitoneally with Na thiopental (40 mg/kg body weight), and then the cage was submerged for one minute in a basin of water (50 × 150 × 50 cm) filled with 50 L of the collected freshwater and saltwater, respectively, with breathing allowed for another minute, this process was repeated until death.

### Freshwater postmortem submersion group (FPS) and saltwater postmortem submersion group (SPS)

Rats were anaesthetised with 40 mg/kg body weight Na thiopental injected intraperitoneal, euthanised by cervical dislocation, and then dipped in fresh and saltwater, respectively.

After the autopsy, the rats' lungs in each group were dissected; the left lungs were prepared for histological and immunohistochemical inspection, while the right lungs were prepared for other biochemical studies.

### Biochemical analysis

#### Sample collection

Tissue homogenates: the tissues were chopped into small pieces and rinsed in ice-cold PBS (0.01 M, pH = 7.4) to completely remove blood. After weighing the tissue pieces, a glass homogenizer on ice was used to homogenize them in PBS (tissue weight (g): PBS (mL) volume = 1:9). Sonicate the suspension with an ultrasonic cell disrupter or subject it to freeze–thaw cycles to break down the cells further. The homogenates were then centrifuged for 5 min at 5000 × g to obtain the supernatant.

#### Measurement of lung oxidative stress/antioxidant biomarkers

Biochemical estimation of lung lipid peroxides done with the use of thiobarbituric acid by reacting with malondialdehyde (MDA), resulting in a pink product whose absorbance can be determined at 534 nm [14].

The Griess reaction, in which nitrite reacts with a solution containing naphthyl ethylenediamine and sulfanilamide to produce a brilliant reddish-purple hue measured at 540 nm, was used to calculate the total nitrite/nitrate (NOx) content of the lung tissue [15].

The Nishikimi and Yogi [16] approach was utilised to determine the superoxide dismutase enzyme (SOD) concentration. The basis of this test is that SOD can inhibit nitroblue tetrazolium dye reduction by phenazine methosulfate, and the enzyme activity was determined using a spectrophotometer at 560 nm.

Reducing 5'-dithiobis-2-nitrobenzoic acid to a yellow hue using Beutler's [17] technique is a chemical approach for measuring the quantity of reduced glutathione (GSH) in lung tissue, the absorbance of a reduced chromogen at 405 nm shows a direct correlation with the concentration of GSH.

#### Assessment of VCAM-1, COX-2 levels in the lung using ELISA assay

VCAM-1 and COX-2 levels in the lung were tested using ELISA kits according to the producer's instructions. Catalog No; E-EL-R106196T and CSB-E13399r, respectively.

The quantitative sandwich enzyme immunoassay method was used in this assay. COX-2 and VCAM-1 specific antibodies have been pre-coated onto a microplate. Once the standards and samples were pipetted into the wells, any marker present was bound by the immobilized antibody. Any unbound substances were removed, and then a biotin-conjugated antibody specific to each marker was added to the wells. After washing, avidin-conjugated Horseradish Peroxidase (HRP) was added to the wells. The wash was repeated to remove any unbound avidin-enzyme reagent. After that, a substrate solution was added to the wells, and colour developed in proportion to the amount of each marker bound in the initial step. The intensity of the colour was measured after the colour development was stopped. The results were calculated by the professional software "Curve Expert" to make a standard curve.

#### Relative measurement of NF-kB and iNOS gene expression using (RT-PCR)

TRI REAGENT solution (Ambion, Warrington, UK, Cat no: TR 118) was used to extract total RNA from 100 μg of rat lungs. The following primers were employed in a thermal cycler (Applied Biosystems GeneAmp® 5700 fast, Cambridge LTD., UK) with 5 μg of total RNA according to the kit's instructions (Promega GoTaq® 1-Step RT-qPCR syber green with ROX Vial Cat no: 53711–5399). The Nanodrop was used to ascertain the RNA's concentration and purity.

After an initial denaturation at 95 °C for 10 min, PCR amplification was carried out using a cycling protocol consisting of 40 cycles (denaturation at 95 °C for 10 s, annealing and extension at 72 °C for 30 s). The levels of gene expression in each sample were compared to those in a control group. The comparative threshold cycle approach (Ct) was used to determine the relative expression levels of the NF-kB and iNOS genes [18]. The GADPH gene was utilised as the reference point for all values.

The primers used were:

**NF-KB gene;** F: 5′- GAG AAG AAC AAG AAA TCC TAC CCA C, 3′

R: 5′- TCC ATT TGT GAC CAA CTG AAC G -3′

**iNOS gene**; F: 5′-CAC CAC CCT CCT TGT TCA AC,3′

R: 5′-CAA TCC ACA ACT CGC TCC AA-3′.

**GADPH gene**; F: 5′- GTA TTG GGC GCC TGG TCA CC -3′,

R: 5′- CGC TCC TGG AAG ATG GTG ATG G -3.

### Histopathological examination

#### Light microscopic study (H&E)

Left lung lobes tissue samples were processed into paraffin blocks straight after being fixed in a 10% buffered formalin solution. Hematoxylin and eosin (H&E) stains were used on serial slices of tissue (5–7 µm) [19]. Two blinded histologists evaluated the histological and immunohistochemical slides for the experimental groups.

To determine and score the intensity of the lung injury. Thickened interalveolar septum, vascular congestion, and infiltration of inflammatory cells were used to compare the histopathological alterations among the experimental groups. Damage severity was measured and categorised as follows: Scores of—were deemed normal, + mild, +  + moderate, and +  +  + severe [20].

#### Immunohistochemistry

Anti-caspase 3 (CAT#ab32351) and Anti-tumour Necrosis Factor Alpha (TNF-α) (CAT#ab220210) were purchased from Abcam, Egypt. Anti-cyclooxygenase 2 (COX 2) (CAT#bs-10411R-TR; Bioss; Egypt), Anti-iNOS (CAT#GB11119; Servicebio, Egypt) and anti-nuclear factor kappa B (NF-κB) (CAT#bs-20159R-TR; Bioss; Egypt) antibodies were also used. All antibodies were utilized in immunohistochemistry experiments as directed by the respective manufacturers.

Sections were deparaffinised, rehydrated, and then subjected to a 20-min antigen retrieval in EDTA buffer in the microwave. Sections were immersed in 0.01 per cent hydrogen peroxide for 10–15 min to inhibit endogenous peroxidase activity. Anti-caspase 3 (1:200) and anti-TNF-α (1:100) polyclonal primary antibodies were then used to incubate sections at 4 °C overnight and, after that, incubation for 30 min with the secondary antibody. The sections were then cleaned and stained with DAB solution for 10 min after being incubated with horseradish peroxidase Envision kit (DAKO) for 20 min. Hematoxylin counterstaining, dehydration, clearing, and mounting were done after sections had been washed in PBS).

Light microscopic images were taken by BX51-microscope and linked to the computer programmed with software LC—micro-application. The image j analysis—software assessed caspase 3, TNF alpha, COX 2 and iNOS immunoreactivity percentages [21] in randomly chosen nine non-overlapping fields at magnification × 400 from each animal in each group. Also number of NF-κB positive nuclei was calculated in randomly chosen nine non-overlapping fields at magnification × 400 from each animal in each group.

### Statistical analysis

Graph Pad Prism was used for the statistical analysis of the data (version 8.01 for Windows, Graph Pad Software, San Diego, California, USA, www.graphpad.com). We calculated the mean and the standard error of the mean. Values were expressed using the mean and the standard error of the mean. Multiple comparisons were made using the one-way ANOVA, then the Tukey-Kramar post hoc test, and we used the student t-test to see whether there were statistically significant differences between each two groups; P-values less than 0.05 are considered statistically significant.

### Sample size calculation

A sample size of 9 rats in each group was determined to provide 80% power for a one-way ANOVA test at the level of 0.05 significance using G Power 3.1 9.2 software.
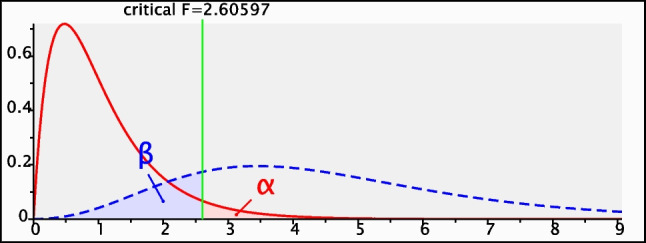


Test family: F test

Statistical test: ANOVA: Fixed effect, omnibus, one-way.

Type of power analysis: Apriori: Compute required sample size- given α, power, and effect size.

Input parameters:

- Effect size: 0.55
- α err prob: 0.05
- Power (1-β): 0.8

Number of groups: 5

Output parameters:

- Noncentrality parameter: 13.6
- Critical F: 2.6
- Sample size per group: 9
- Total sample size: 45
- Actual power: 0.8060

## Results

### Lung oxidative stress/antioxidant biomarkers

The levels of MDA & NOx in the lungs were significantly elevated in the drowned groups (FD and SD) compared with the control group (C) and postmortem submersion groups (FPS and SPS), with the highest level found in the lungs of the (SD) group (Table 1).

**Table 1:** Means ± SD lung oxidative stress/antioxidant biomarkers

Groups	MDA (nmol/g tissue)	NOx (µmol/g tissue)	SOD (U/g tissue)	GSH (mmol/g tissue)
C-group	0.79 ± 0.11^**bd**^	1.25 ± 0.13 ^**bd**^	2.5 ± 0.1^**bd**^	2.96 ± 0.17 ^**bd**^
FD-group	1.65 ± 0.11^**acde**^	3.01 ± 0.19^**acde**^	1.3 ± 0.07^**acde**^	2.08 ± 0.12^**acde**^
FPS-group	0.83 ± 0.11^**bd**^	1.27 ± 0.18^**bd**^	2.5 ± 0.13^**bd**^	2.8 ± 0.14^**bd**^
SD-group	2.25 ± 0.16^**abce**^	4.3 ± 0.24^**abce**^	0.94 ± 0.06^**abce**^	1.5 ± 0.07^**abce**^
SPS-group	0.83 ± 0.097^**bd**^	1.4 ± 0.23^**bd**^	2.4 ± 0.12^**bd**^	2.8 ± 0.16^**bd**^

Results represent the mean ± SEM (9 rats /group). ^a^ significant difference from the C-group, ^b^ significant difference from the FD-group, ^c^ significant difference from the FPS-group, ^d^ significant difference from the SD-group, ^e^ significant difference from the SPS-group. *P* ≤ 0.05. *MDA* Malondialdhyde; *NOx* Total nitrite/nitrate; *SOD* Superoxide dismutase; *GSH* Reduced glutathione

Lung antioxidant biomarkers (SOD and GSH) levels were significantly decreased in both drowned groups (FD and SD), with significantly more depletion in the (SD) group when compared to the other three groups (C, FPS, and SPS) (Table 1).

There were no statistically significant differences in MDA, NOx, SOD, and GSH levels among the control group and postmortem submersion groups (FPS and SPS) (Table 1).

### Levels of VCAM-1 and COX-2 in lung tissues

Drowned groups (FD and SD) showed a significant increase in pulmonary levels of (VCAM-1 and COX-2) in comparison to the other groups (C, FPS, and SPS). The levels of these parameters were significantly higher in the (SD) group than in the (FD) group. They also revealed no significant difference among the non-drowned groups (C, FPS, and SPS) (Table 2).

**Table 2:** Means ± SD of VCAM-1 and COX2 in lung tissue

Groups	VCAM-1 (pg/ml tissue)	COX2 (ng/ml tissue)
C-group	10.97 ± 0.8^**bd**^	0.98 ± 0.13 ^**bd**^
FD-group	26.8 ± 0.7^**acde**^	1.7 ± 0.07^**acde**^
FPS-group	11.2 ± 0.9^**bd**^	1.07 ± 0.19^**bd**^
SD-group	32.08 ± 1.85^**abce**^	2.1 ± 0.13^**abce**^
SPS-group	10.83 ± 1.05^**bd**^	0.94 ± 0.13^**bd**^

Results represent the mean ± SEM (9 rats /group). ^a^ significant difference from the C-group, ^b^ significant difference from the FD-group, ^c^ significant difference from the FPS-group, ^d^ significant difference from the SD-group, ^e^ significant difference from SPS-group. *P* ≤ 0.05. VCAM-1: vascular cell adhesion protein 1; COX2: cyclooxygenase-2

### NF-kB and iNOS mRNA gene expression

The NF-kB and iNOS genes showed significantly highest expression in the lungs of the (SD) group, followed by the (FD) group. However, they didn't reveal any significant difference among the other groups (C, FPS, and SPS) (Table 3).

**Table 3:** Means ± SD NF-κB and iNOS mRNA gene expression

Groups	NF-κB	iNOS
C-group	0.75 ± 0.05^**bd**^	0.57 ± 0.06 ^**bd**^
FD-group	1.8 ± 0.08^**acde**^	1.5 ± 0.05^**acde**^
FPS-group	0.86 ± 0.08^**bd**^	0.62 ± 0.07^**bd**^
SD-group	2.4 ± 0.12^**abce**^	1.9 ± 0.13^**abce**^
SPS-group	0.82 ± 0.08^**bd**^	0.64 ± 0.08^**bd**^

Results represent the mean ± SEM (9 rats /group). ^a^ significant difference from the C-group, ^b^ significant difference from the FD-group, ^c^ significant difference from the FPS-group, ^d^ significant difference from the SD-group, ^e^ significant difference from the SPS-group. *P* ≤ 0.05. *NF-κB* Nuclear factor kappa B; *iNOS* Inducible nitric oxide synthase enzyme

### Histopathological examination

#### The Hematoxylin and Eosin (H&E) results and histopathological scoring

**Fig. 1:**
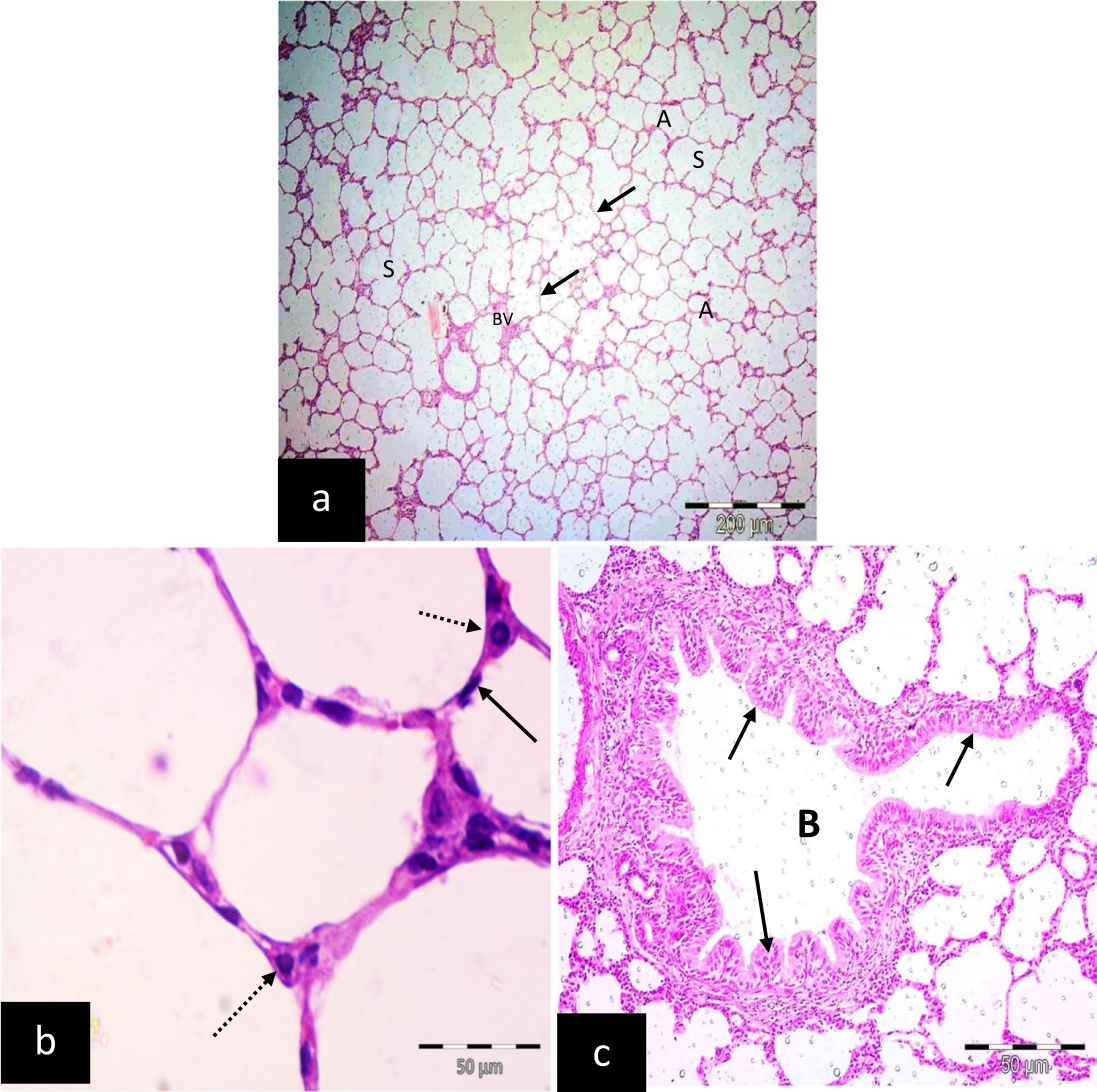
Illustrative photomicrographs of the lung of adult male albino rats of the control group; **a** showing alveoli (A), alveolar sacs (S), and blood vessels(B.V). Notice thin inter-alveolar septa (arrows). **b** showing interalveolar septum formed of a single layer of cells: pneumocyte type I (arrow) and pneumocyte type II (dotted arrows). **c** showing bronchiole (B) lined with simple columnar epithelium (arrows). (H&E; a X 100, scale bar = 200 μm; b, c X 400, scale bar = 50 μm)

The control group (C) showed normal alveoli, alveolar sacs, and blood vessels. Thin inter-alveolar septa appeared (Fig. 1a). The interalveolar septum was formed of a single layer of cells: pneumocyte type I and pneumocyte type II (Fig. 1b). The bronchiole is lined with simple columnar epithelium (Fig. 1c).

**Fig. 2:**
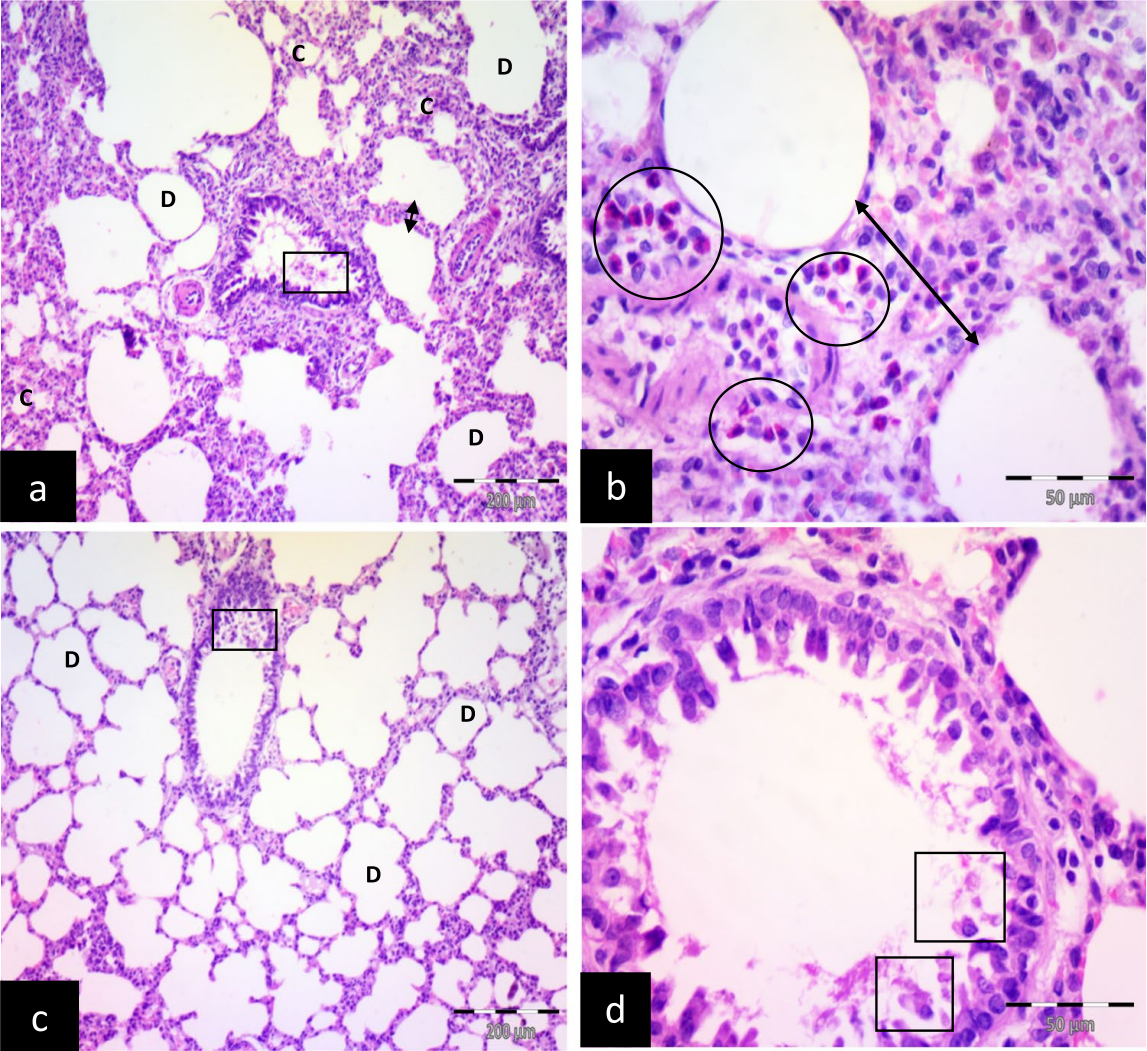
Illustrative photomicrographs of the lung of adult male albino rats from; **a**, **b** freshwater-drowned group (FD), showing thickening of the inter-alveolar septa (double head arrows) with inflammatory cellular infiltration (circles). Some alveoli are collapsed (c), and others are dilated (D). Notice: exfoliated epithelial cells (square) inside the bronchioles. **c**, **d** freshwater postmortem submersion group (FPS), showing dilated alveoli (D) and exfoliated epithelial cells (squares) inside the bronchioles. (H&E; a, c, X 100, scale bar = 200 μm; b, d X 400, scale bar = 50 μm)

The freshwater-drowned group (FD) revealed the inter-alveolar septa thickening with inflammatory cellular infiltration. Some alveoli collapsed, and others dilated. Exfoliated epithelial cells inside the bronchioles were also noticed (Fig. 2a, b).

Freshwater postmortem submersion group (FPS) revealed dilated alveoli and exfoliated epithelial cells inside the bronchioles (Fig. 2c, d).

**Fig. 3:**
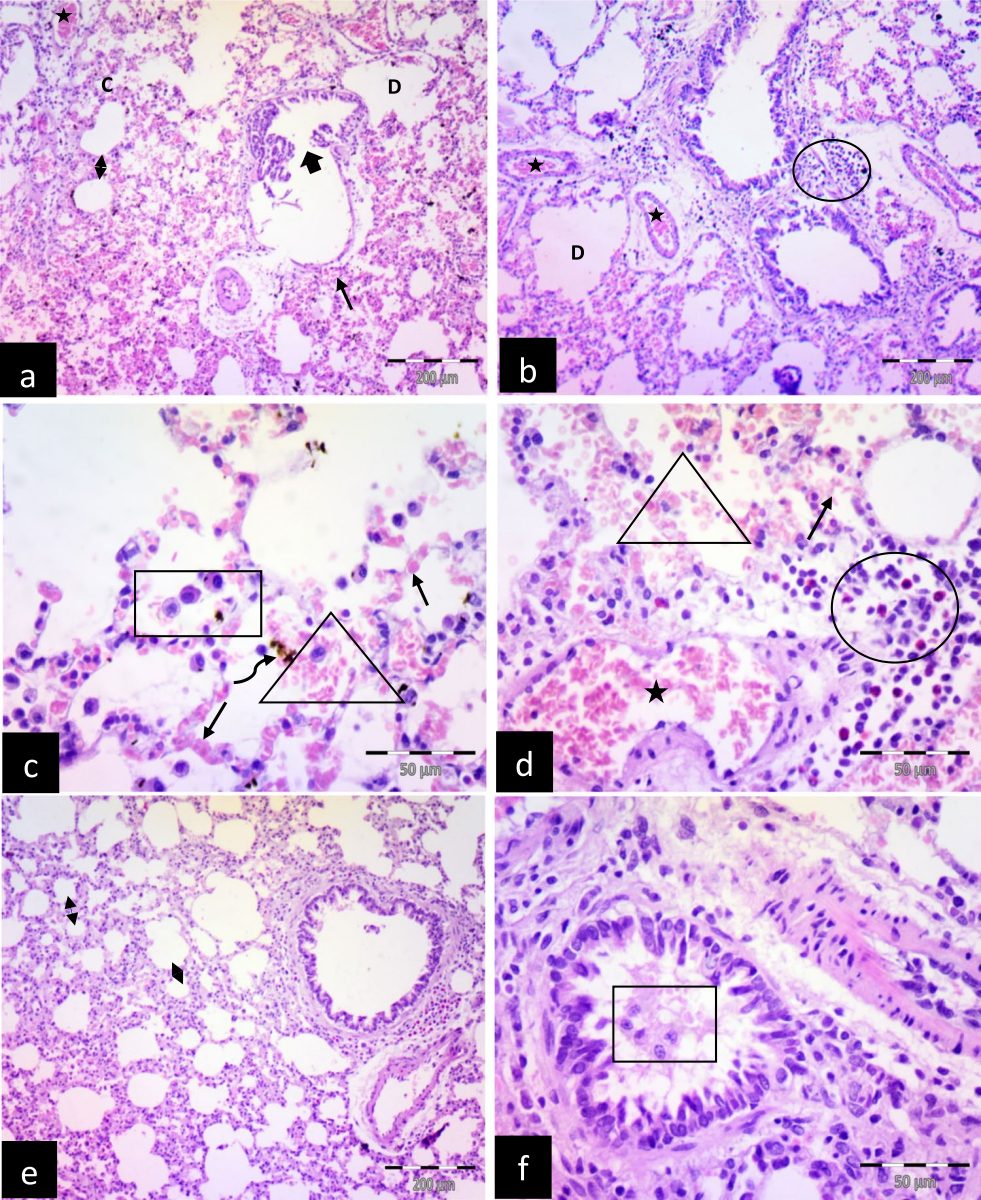
Illustrative photomicrographs of the rats' lungs from different experimental groups in (**a**, **b**, **c**, **d**) saltwater-drowned group (SD); showing thickening of the inter-alveolar septa (double head arrow) with congested blood vessels (stars), extravasated RBCs in the interalveolar septa (thin arrows) and inside the alveoli (triangles) with inflammatory cellular infiltration (circles). Some alveoli are collapsed (c), and others are dilated (D). Notice: destructed bronchioles (thick arrow), hemosiderin-laden macrophages (curved arrow), and intra-alveolar lymphocytes (square). **e**, **f** Saltwater postmortem submersion group (SPS): Showed thickening of the inter-alveolar septa (double head arrows) and exfoliated epithelial cells (square) inside the bronchioles. (H&E; a, b, e X 100, scale bar = 200 μm; c, d, f X 400, scale bar = 50 μm)

The saltwater-drowned group (SD) showed interalveolar septa thickening with congested blood vessels and extravasated RBCs in the interalveolar septa and inside the alveoli with inflammatory cellular infiltration. Some alveoli collapsed, and others dilated. Destructed bronchioles, hemosiderin-laden macrophages, and intra-alveolar lymphocytes were also observed (Fig. 3a, b, c, d).

Saltwater postmortem submersion group (SPS) revealed thickening of the inter-alveolar septa and exfoliated epithelial cells inside the bronchioles (Fig. 3 e, f).

Lung injury in the form of alveolar haemorrhage, vascular congestion, and inflammation was maximum in the (SD) group, followed by the (FD) group (Table 4).

**Table 4:** The histopathological scoring of changes found in the lungs of the different experimental groups

Histological changes	Group C	Group FD	Group FPS	Group SD	Group SPS
Thickened interalveolar septum	-	+ +	-	+ + +	+ +
Vascular congestion	-	+ +	-	+ + +	-
Inflammatory cellular infiltrate	-	+ +	-	+ + +	-

The structural changes of tissue were assessed according to the degree of thickened interalveolar septum, vascular congestion, and inflammatory cells infiltration. The severity of lesions was graded as the following: score − was considered normal, score + mild, score +  + moderate, and score +  +  + severe [20]

#### Immunohistochemical results for anti-caspase 3 antibodies

**Fig. 4:**
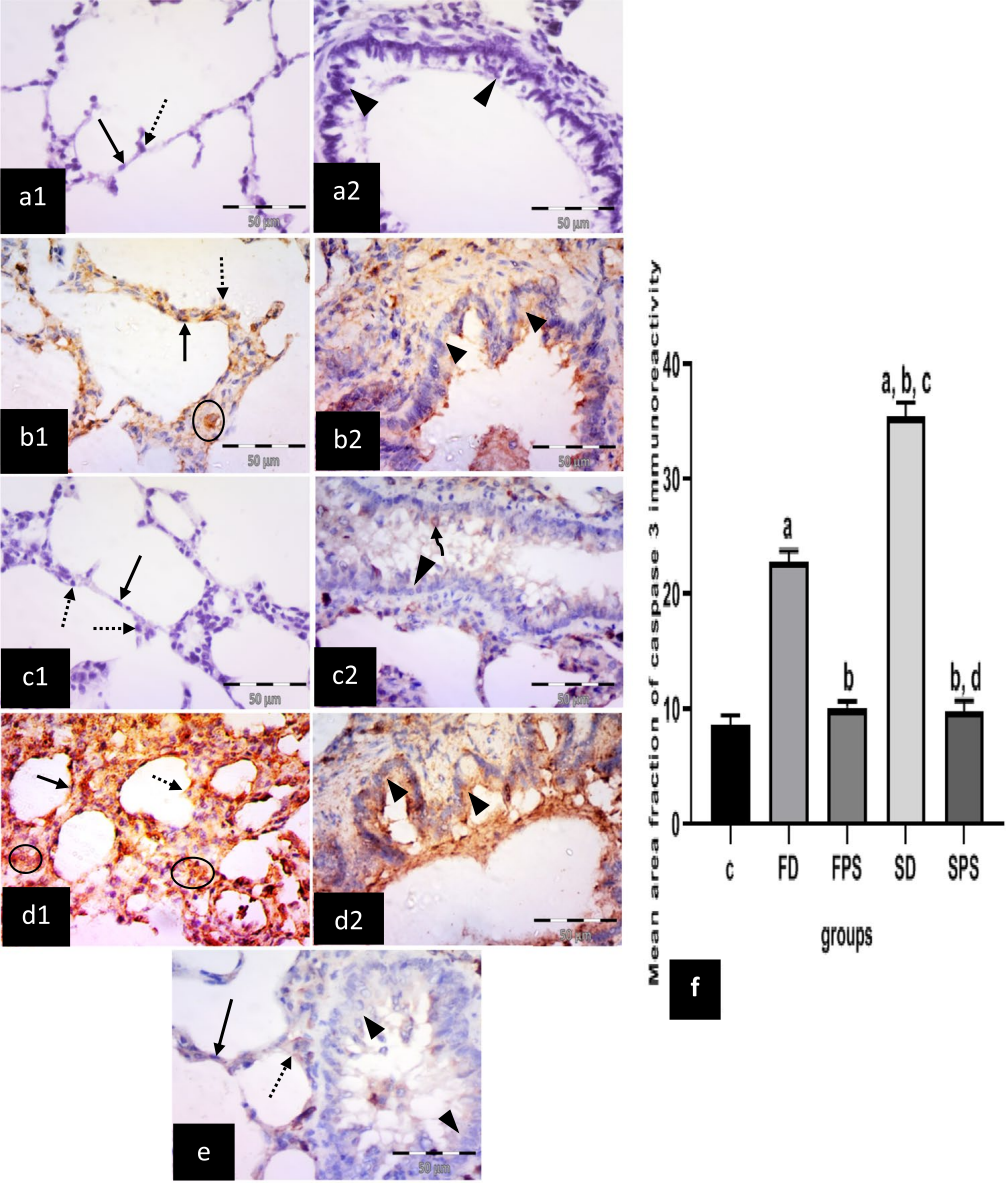
Illustrative photomicrographs of the lung from all experimental groups in adult male albino rats immunolabelled for caspase 3 in; **a1, 2** control group(C); showing negative immuno-expression in type I pneumocytes (arrow), type II pneumocytes (dotted arrow) and epithelial lining of the bronchiole (arrow heads). **b1, 2** freshwater-drowned group (FD), revealing many cells with strong positive cytoplasmic (circle) expression, most properly macrophages in the interalveolar space. Strong positive cytoplasmic expression in pneumocytes type I (arrow) and type II (dotted arrow). Moderate positive cytoplasmic expression in the bronchiolar epithelium (arrowhead) is also noticed. **c1, 2** freshwater postmortem submersion group (FPS), showing negative cytoplasmic expression in type I pneumocytes (arrow) or type II pneumocytes (dotted arrows). Notice negative (head arrow) or faint (curved arrow) cytoplasmic expression in the bronchiolar epithelium. **d1, 2** saltwater-drowned group(SD), showing numerous cells with strong positive cytoplasmic expression in the interalveolar septum (circles). Notice strong cytoplasmic expression in type I (arrow) and type II (dotted arrow) pneumocytes and strong cytoplasmic expression in the bronchiolar epithelium (arrowheads). **e** saltwater postmortem submersion group (SPS), revealing faint cytoplasmic expression in type I pneumocytes (arrow) and type II pneumocytes (dotted arrow). Notice negative or faint cytoplasmic expression in the bronchiolar epithelium (arrowheads) (*n* = 9) (immunohistochemistry for caspase 3 × 400, scale bar = 50 μm). **f** Mean area fraction of caspase 3 immunoreactivity, ^a^ significant difference from the control group, ^b^ significant difference from (FD) group, ^c^ significant difference from (FPS) group, d significant difference from (SD) group. *P* < 0.05. Values are expressed as mean ± SEM (*n*=9)

The control group showed negative immuno-expression in type I pneumocytes, type II pneumocytes and epithelial lining of the bronchiole (Fig. 4 a1, 2). The (FD) group revealed many cells with positive cytoplasmic expression most properly macrophages. Strong positive cytoplasmic expression in pneumocytes type I and type II was revealed. Moderate positive cytoplasmic expression in the bronchiolar epithelium was also noticed (Fig. 4 b1, 2). The (FPS) group showed negative cytoplasmic expression in type I pneumocytes or type II pneumocytes. Negative or faint cytoplasmic expression in the bronchiolar epithelium was noticed (Fig. 4 c1, 2). The (SD) group revealed numerous cells with strong positive cytoplasmic expression in the interalveolar septum, most properly macrophages. Strong cytoplasmic expression in type I and type II pneumocytes was noticed. Strong cytoplasmic expression in the bronchiolar epithelium was also revealed. The (SPS) group revealed faint cytoplasmic expression in type I pneumocytes and type II pneumocytes. Negative or faint expression in the bronchiolar epithelium was revealed (Fig. 4 e).

#### Immunohistochemical results for anti-TNF-*α* antibodies

**Fig. 5:**
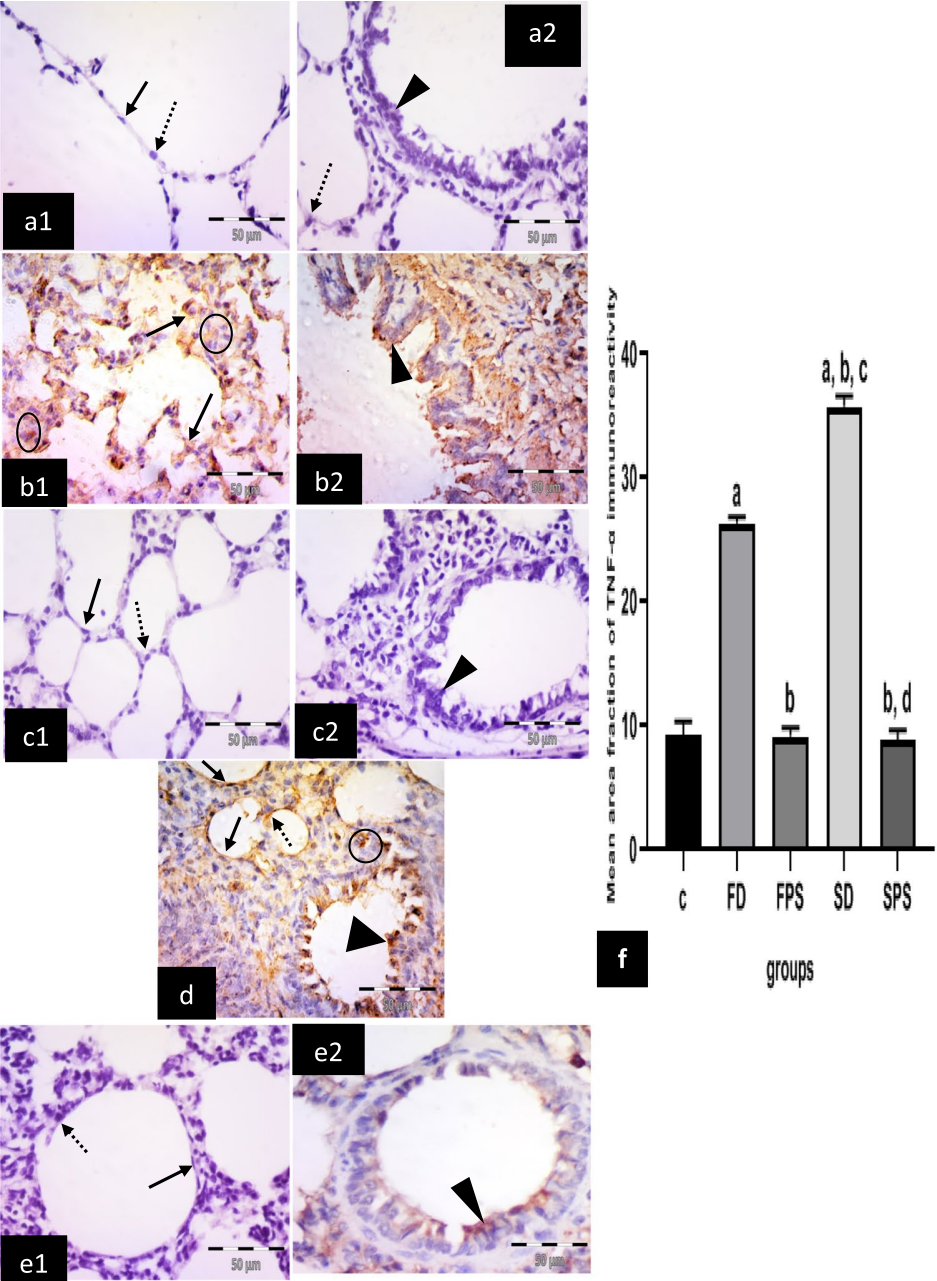
Illustrative photomicrographs of the lung from all experimental groups in adult male albino rats immunolabelled for TNF-α in (**a1, 2**) control group (C); showing negative immuno-expression in type I pneumocytes (arrow), type II pneumocytes (dotted arrow) and epithelial lining of the bronchiole (arrowhead). **b1, 2** freshwater-drowned group (FD), revealing many cells with positive cytoplasmic expression (arrows) in the alveolar lining, most properly pneumocytes type II (arrows) and positive cytoplasmic expression in the bronchiolar epithelium (arrowhead) and cells in the interalveolar septum (circles). **c1, 2** freshwater postmortem submersion group (FPS), showing negative cytoplasmic expression in type I (arrow) and type II (dotted arrow) pneumocytes. Notice negative (head arrow) cytoplasmic expression in bronchiolar epithelium. **d** saltwater-drowned group (SD), showing numerous cells with positive cytoplasmic expression in cells lining the alveoli, either type I pneumocytes (arrows) or type II pneumocytes (dotted arrow). Notice cells with positive cytoplasmic expression in the bronchiolar epithelium (arrowhead) and the interalveolar septum (circle). **e1, 2** saltwater postmortem submersion group (SPS), revealing negative cytoplasmic expression in cells lining the alveoli, either type I (arrow) or type II (dotted arrow) pneumocytes. Notice some cytoplasmic expression in the bronchiolar epithelium (arrowhead). (*n* = 9) (immunohistochemistry for TNF-α × 400, scale bar = 50 μm). **f** Mean area fraction of TNF-α immunoreactivity, a significant difference from the control group, b significant difference from (FD) group, c significant difference from (FPS) group, d significant difference from (SD) group. *P* < 0.05. Values are expressed as mean ± SEM (*n* = 9)

The control group showed negative immuno-expression in type I pneumocytes, type II pneumocytes and epithelial lining of the bronchiole (Fig. 5 a1, a2). The (FD) group revealed many cells with positive cytoplasmic expression in the alveolar lining, most properly pneumocytes type II and positive cytoplasmic expression in bronchiolar epithelium and in cells in the interalveolar septum (Fig. 5 b1, b2). The (FPS) group showed negative cytoplasmic expression in type I and type II pneumocytes. Negative cytoplasmic expression in bronchial epithelium was also noticed (Fig. 5 c1, c2). The (SD) group revealed numerous cells with positive cytoplasmic expression in cells lining the alveoli, either type I pneumocytes or type II pneumocytes. Cells with positive cytoplasmic expression in the bronchiolar epithelium and the interalveolar septum were noticed (Fig. 5 d). The (SPS) group revealed negative cytoplasmic expression in cells lining the alveoli either types I or II pneumocytes. Some expression in bronchiolar epithelium was revealed (Fig. 5 e1, 2).

#### Immunohistochemical results for anti-COX 2 antibodies

**Fig. 6:**
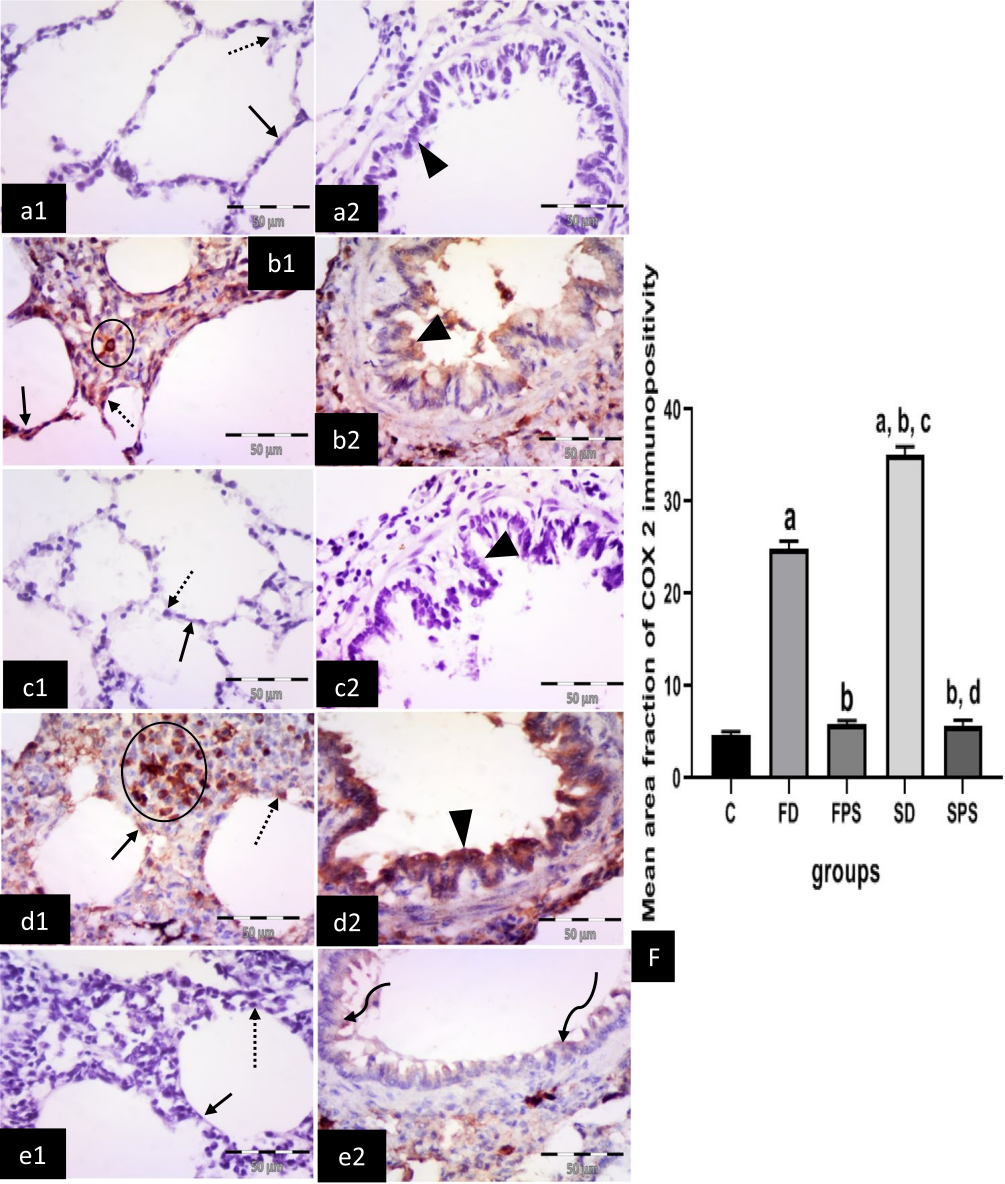
Illustrative photomicrographs of the lung from all experimental groups in adult male albino rats immunolabelled for COX 2 in (**a1, 2**) control group (C): showing negative immuno-expression in type I pneumocytes (arrow), type II pneumocytes (dotted arrow) and epithelial lining of the bronchiole (arrowhead). **b1, 2** freshwater-drowned group (FD) revealing many cells with positive cytoplasmic expression in the alveolar lining, either type I (arrow) or type II (dotted arrow) pneumocytes, in the bronchiolar epithelium (arrowhead) and connective tissue cells in the interalveolar septum (circle). **c1, 2** freshwater postmortem submersion group (FPS) and (**e1, 2**) saltwater postmortem submersion group (SPS), showing negative cytoplasmic expression in type I (arrows) and type II (dotted arrows) pneumocytes. Notice negative (head arrow) or faint (curved arrows) cytoplasmic expression in the bronchiolar epithelium. **d1, 2** saltwater-drowned group (SD), showing numerous cells with positive strong cytoplasmic expression in cells lining the alveoli, either type I (arrow) or type II (dotted arrow) pneumocytes. Notice strong positive cytoplasmic expression in the bronchiolar epithelium (arrowhead) and connective tissue cells in the interalveolar septum (circle). (*n* = 9) (immunohistochemistry for COX 2 × 400, scale bar = 50 μm). **(f)** Mean area fraction of COX 2 immunoreactivity, ^a^ significant difference from the control group, ^b^ significant difference from (FD) group, ^c^ significant difference from ( FPS) group, ^d^ significant difference from (SD) group. *P* < 0.05. Values are expressed as mean ± SEM (*n* = 9)

The control group showed negative immuno-expression in type I pneumocytes, type II pneumocytes and epithelial lining of the bronchiole (Fig. 6 a1, a2). The (FD) group revealed many cells with positive cytoplasmic expression in the alveolar lining, either type I or II pneumocytes, in the bronchiolar epithelium and connective tissue cells in the interalveolar septum (Fig. 6 b1, 2). The (FPS) and the (SPS) groups revealed negative cytoplasmic expression in type I and type II pneumocytes. Negative or faint cytoplasmic expression in the bronchiolar epithelium was noticed (Fig. 6 c1, 2; e1, 2). The (SD) group revealed numerous cells with positive strong cytoplasmic expression in cells lining the alveoli, either type I or type II pneumocytes. Strong cytoplasmic expression in bronchiolar epithelium and connective tissue cells in the interalveolar septum were noticed (Fig. 6 d1,2).

#### Immunohistochemical results for anti-iNOS antibodies

**Fig. 7:**
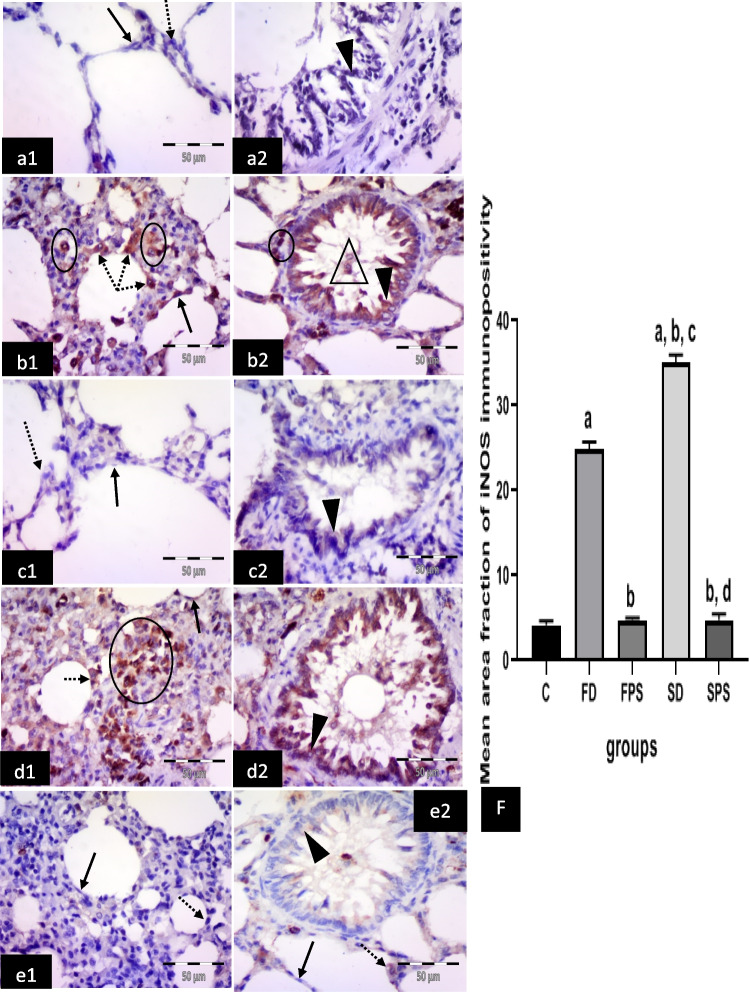
Illustrative photomicrographs of the lung from all experimental groups in adult male albino rats immunolabelled for iNOS 2 in (**a1, 2**) control group (C): showing negative immuno-expression in type I pneumocytes (arrow), type II pneumocytes (dotted arrow) and epithelial lining of the bronchiole (arrowhead). **b1, 2** freshwater-drowned group (FD), revealing many cells with strong positive cytoplasmic expression in the alveolar lining, either type I (arrow) or type II (dotted arrows) pneumocytes, in connective tissue cells in the interalveolar septum (circles), in the bronchiolar epithelium (arrowhead) and cells in the bronchiolar lumen (triangle). **c1, 2** freshwater postmortem submersion group (FPS), showing negative cytoplasmic expression in type I (arrow) and type II (dotted arrow) pneumocytes. Notice very faint (head arrow) cytoplasmic expression in the bronchiolar epithelium. **d1, 2** saltwater-drowned group (SD), showing numerous cells with strong positive cytoplasmic expression in the lining of the alveoli, either type I (arrow) or type II pneumocyte (dotted arrow). Notice strong positive cytoplasmic expression in the bronchiolar epithelium (arrowhead) and connective tissue cells in the interalveolar septum (circle). **e1, 2** saltwater postmortem submersion group (SPS), revealing negative or faint cytoplasmic expression in cells lining the alveoli, either type I (arrows) or type II pneumocyte (dotted arrows) and in the bronchiolar epithelium (arrowhead). (*n* = 9) (immunohistochemistry for iNOS × 400, scale bar = 50 μm). **f** Mean area fraction of iNOS immunoreactivity, ^a^ significant difference from the control group, ^b^ significant difference from (FD)group, ^c^ significant difference from (FPS) group, ^d^ significant difference from (SD) group. *P* < 0.05. Values are expressed as mean ± SEM (*n* = 9)

The control group revealed negative immuno-expression in type I pneumocytes, type II pneumocytes and epithelial lining of the bronchiole (Fig. 7 a1, 2). The (FD) group showed many cells with strong positive cytoplasmic expression in the alveolar lining, either type I or type II pneumocytes, in connective tissue cells in the interalveolar septum, in the bronchiolar epithelium and cells in the bronchiolar lumen (Fig. 7 b1,2). The (FPS) group showed negative cytoplasmic expression in type I and type II pneumocytes. Very faint cytoplasmic expression in the bronchiolar epithelium was noticed (Fig. 7 c1, 2). The (SD) group showed numerous cells with strong positive cytoplasmic expression in the lining of the alveoli, either type I or type II pneumocytes. Strong positive cytoplasmic expression in bronchiolar epithelium and connective tissue cells in the interalveolar septum was noticed (Fig. 7 d1, 2). The (SPS) group revealed negative or faint cytoplasmic expression in cells lining the alveoli, either type I or type II pneumocytes and bronchiolar epithelium (Fig. 7 e1, 2).

#### Immunohistochemical results for anti-NF-κB antibodies

**Fig. 8:**
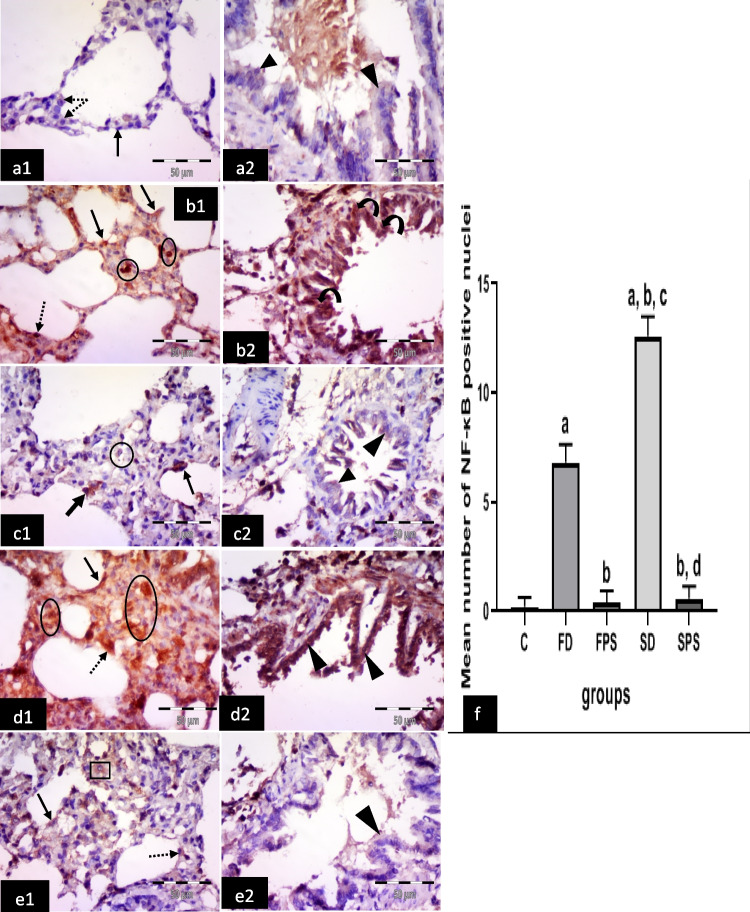
Illustrative photomicrographs of the lung from all experimental groups in adult male albino rats immunolabelled for NF-κB2 in; **a1, 2** control group (C); showing negative or faint cytoplasmic and negative nuclear expression in type I pneumocytes (arrow), type II pneumocytes (dotted arrows) and epithelial lining of a bronchiole (arrow heads). **b1, 2** freshwater-drowned group (FD), revealing many cells with strong positive cytoplasmic (arrows) or nuclear and cytoplasmic (dotted arrow) expression in the alveolar lining, strong nuclear expression in connective tissue cells in the interalveolar septum (circles) and strong nuclear and cytoplasmic expression in the bronchiolar epithelium (curved arrows). **c1, 2** freshwater postmortem submersion group (FPS), showing some cells with positive cytoplasmic expression in the alveolar lining (arrows) and moderate cytoplasmic expression in the bronchiolar epithelium (arrowheads) and cells in the interalveolar septum (circle). **d1, 2** saltwater-drowned group (SD), showing numerous cells with strong positive cytoplasmic and nuclear expression in the alveoli (type I (arrow) and type II (dotted arrow) pneumocytes), in connective tissue cells in the interalveolar septum (circle) and epithelium lining of the bronchiole (arrowheads). **e1, 2** saltwater postmortem submersion group (SPS), revealing positive cytoplasmic expression in cells lining the alveoli, either type I (arrow) or type II pneumocyte (dotted arrow) and cells in the interalveolar septum (square) and faint cytoplasmic expression in the bronchiolar epithelium (arrowhead). (*n* = 9) (immunohistochemistry for NF-κBx 400, scale bar = 50 μm). **f** Mean number of NF-κB positive nuclei, ^a^ significant difference from the control group, ^b^ significant difference from (FD) group, ^c^ significant difference from (FPS) group, ^d^ significant difference from (SD) group. *P* < 0.05. Values are expressed as mean ± SEM (*n* = 9)

The control group showed negative or faint cytoplasmic and nuclear expression in type I pneumocytes, type II pneumocytes and epithelial lining of the bronchiole (Fig. 8 a1, a2). The (FD) group revealed many cells with strong positive cytoplasmic or nuclear and cytoplasmic expression in the alveolar lining, strong nuclear expression in connective tissue cells in the interalveolar septum and strong nuclear and cytoplasmic expression in bronchiolar epithelium (Fig. 8 b1, b2). The (FPS) group showed some cells with positive cytoplasmic expression in the alveolar lining and moderate cytoplasmic expression in bronchiolar epithelium and cells in the interalveolar septum (Fig. 8 c1, c2). The (SD) group showed numerous cells with strong positive cytoplasmic and nuclear expression in the alveoli (type I and type II pneumocytes), in connective tissue cells in the interalveolar septum and epithelium lining of the bronchiole (Fig. 8 d1, 2). The (SPS) group revealed positive cytoplasmic expression in cells lining the alveoli, either type I or type II pneumocytes and cells in the interalveolar septum and faint cytoplasmic expression in the bronchiolar epithelium (Fig. 8 e1, e2).

Statistically, there was a significant increase in the immuno-expression of caspase 3, TNF*-*α, COX 2, iNOS and intranuclear NF-κB in the (SD) group, followed by the (FD) group, respectively. However, it revealed non-significant differences among the non-drowned groups (C, FPS and SPS) (Figs. 4f, 5f, 6f, 7f, and 8f).

## Discussion

The majority of the pathological findings linked to drowning are not pathognomonic, so the forensic pathologist has to reach a precise diagnosis; furthermore, distinguishing a body disposed of in water following decease from a drowning victim is still one of the most confusing questions for forensic pathologists, specifically after an extended postmortem period [22]. The purpose of this research was to investigate whether oxidative stress indicators and the NF-KB/iNOS inflammatory pathway could be used to diagnose drowning and distinguish between freshwater and saltwater drowning.

In the current model of drowning, we found that only in drowned groups (FD and SD) pulmonary oxidative/nitrosative stress indicators were augmented, as denoted by the significant rise in MDA and NOx contents in lung tissues with the drop of SOD and GSH concentrations. Moreover, the inflammatory pathway was stimulated and presented with highly significant levels of the tested markers (cytoplasmic & intra nuclear NF-KB, iNOS, COX-2, TNF- α, VCAM-1), which was not produced in postmortem submersion groups (FPS and SPS). These results were reinforced by the histopathological findings and immuno-expression of the apoptotic marker (caspase 3), which was significantly higher in drowned groups. Likewise, Ma et al. [23] demonstrated that saltwater inspiration could increase NO production, inflammatory cytokines release (TNF-α and IL-1beta), and expression of NF-κB and i-NOS in the lungs. Also, Sun et al. [24] revealed that saltwater exposure could significantly increase intracellular reactive oxygen species (ROS) and inflammatory cytokines (IL-6, IL-8, and TNF-α) production, reduce the viability of the cells and induce apoptosis. However, the comparable results did not differentiate saltwater from freshwater drowning or postmortem submersion.

These results are attributed to hypoxia, which induces oxidative stress within the different cells by inhibiting the activity of the cytochrome chain and mitochondrial oxidative phosphorylation that subsequently diminishes adenosine triphosphate molecule production and increases ROS generation. It also impairs the cellular antioxidant system activity [25]. In lung injuries, oxidative stress and undue ROS production are closely correlated to the number of inflammatory cells and the released cytokines. They can trigger apoptosis or even necrosis in severe cases [26, 27].

Moreover, drowning causes lung inflammation and type II alveolar epithelial cell injury since after fluid inhalation, the altered osmotic pressure in the alveoli induces lung oedema, electrolyte, and metabolic changes, and surfactant malfunction resulting in disruption of lung ventilation/perfusion ratio that can finally lead to acute lung injury. Large amounts of inflammatory cytokines and chemokines are then released, which in turn activate neutrophils and set off an inflammatory cascade. Acute lung injury may be promoted or exacerbated by this abnormally agitated inflammatory response [28, 29].

Nuclear factor kappa (NF-κB) is the major transcription factor that orchestrates inflammatory cytokines in lung inflammation [30]. The imperative target genes of NF-κB are iNOS, COX-2, and TNF-α, which are key mediators in the inflammatory process. TNF-α, released early in inflammation, can initiate the infiltration of immune cells into the location of inflammation by activating VCAM-1 and other adhesion molecules. These cytokines, in turn, stimulate NF-κB transcription. The positive feedback is supposed to increase the amplitude of the inflammatory signals and aggravate tissue damage [31]. It is noteworthy that the detection of the intranuclear presence of NF-κB by immunohistological studies verifies NF-κB activation, which was obvious in saltwater-drowned and freshwater-drowned rats in our study.

It also should be mentioned that NO and NF-κB signalling pathways are closely linked as NO modulates NF-κB at different stages of its activation pathway. NF-κB activation is crucial for iNOS and COX-2 gene transcription that are responsible for adjusting NOx levels within the tissues. Thus, NF-κB and its dependent proinflammatory cytokines contribute to lung injury and apoptosis by provoking endothelial cell permeability, promoting neutrophil recruitment, and more cytokine release [23].

Regarding comparing the results of the drowned groups to each other, it was found that drowning in saltwater induced more oxidative stress, inflammation, and tissue damage. That was presented by significantly more affected oxidative stress indicators and significantly higher levels of all assessed inflammatory markers. Moreover, the histopathological examination of lung tissues revealed more congestion, haemorrhage, and inflammatory cell infiltration in the (SD) group. These results are consonant with Rui et al. [32], who revealed that oxidative stress markers in lung tissues were more affected in saltwater drowning than in freshwater drowning, with the inflammation and cytokines (TNF- α and IL-1beta) being more prominent in saltwater drowning.

In the same line, Ahmed & Hussein [30] found that the transcriptional level of NF-κB was highly expressed in the lung tissue of saltwater-drowned rats compared with freshwater-drowned rats. Levels of proinflammatory cytokines (TNF-α, IL-1beta, and IL-6) were significantly elevated in bronchoalveolar lavage fluid of the saltwater-drowned rats as well. Lee et al. [1] examined the role of another inflammatory marker (receptor for advanced glycation end products; RAGE) in determining the drowning media (saltwater and freshwater drowning) and found that RAGE expression in the lungs of saltwater drowned group was significantly raised compared with freshwater drowned group.

The results partially go with Legaz et al. [33], who revealed that seawater drowning had increased the levels of both MDA and GSH in the lungs. They attributed the elevated level of GSH to a response to oxidative stress and MDA production induced by hypoxia. Still, due to rapid death, time was inadequate for consuming antioxidants, including GSH.

The investigated markers were significantly more affected in saltwater drowning, which could be explained by the fact that saltwater inspiration may result in more direct (mechanical asphyxia) as well as indirect damage to lung tissue [34]. It is a cold, hyperosmotic liquid with elevated sodium chloride and calcium concentrations and numerous pathogens. When inspired into the lungs, it draws the fluids from the pulmonary capillaries and surrounding extracellular spaces into the alveolar sac, producing pulmonary oedema, which is three-fold higher in severity than oedema caused by freshwater, in addition to hemoconcentration, hypovolemia, and hypoproteinemia [35].

Aspiration of hypertonic fluids has also been linked to a variety of pathophysiological changes in lung tissue cells, including atrophy in alveolar epithelial and vascular endothelial cells, apoptosis, and breakdown of the blood-gas barrier. Aspiration of saltwater, pathogens incursion, and lung edema may all work together to stimulate neutrophil chemotaxis and the production of plentiful inflammatory cytokines. In addition to ROS generation, neutrophilic activation stimulates the release of certain vasoactive chemicals, which exacerbates the pulmonary damage. Also, seawater aspiration can stimulate alveolar epithelial cells to generate more auto phagosomes, which induces abnormal inflammatory reactions or cellular apoptosis [36].

Meanwhile, freshwater drowning results in different physio-pathological changes that include hypervolemia, pulmonary oedema, hemolysis, hyperkalemia, hyponatremia, and hypoalbuminemia due to the hasty diffusion of hypotonic freshwater into the circulation, with a high risk of ventricular fibrillation that leads to rapid death. So, the acute lung injury and inflammation in saltwater drowning are graver than those that occur during freshwater drowning [30].

## Conclusions

In this research, we analyzed oxidative stress and inflammatory markers in different drowning models, and we concluded that all analyzed biomarkers were significantly more affected in drowned groups than in postmortem submersion groups. Moreover, saltwater significantly induced oxidative stress and inflammation more than freshwater. Therefore, our results provide great evidence that pulmonary oxidative stress biomarkers and NF-KB/iNOS inflammatory pathway could be valuable diagnostic markers in drowning and differentiating it from postmortem submersion. Moreover, they could help forensic experts to distinguish between freshwater drowning and saltwater drowning. Up to our knowledge, this is the first study to evaluate an innovative pathway, NF-KB/iNOS inflammatory pathway, to investigate drowning in forensic practice.

The limitations of our study are that the present outcomes are restricted to the practical application of forensic casework and the absence of forensic autopsy samples. In addition, the influence of postmortem interval on the studied markers is not evaluated. These points should be considered in future research with investigating other markers in different tissues.

## Data Availability

All data analysed in this study are included in the article.
